# Impacts of food consumption on biochemical markers and anthropometric variables of women with metabolic syndrome

**DOI:** 10.1186/s12905-022-02010-7

**Published:** 2022-10-26

**Authors:** Kelly Cristiane Michalichen, Vinícius Muller Reis Weber, Marcos Roberto Queiroga, Daniel Zanardini Fernandes, Clisia Mara Carreira, Edgar Ramos Vieira, Danielle Venturini

**Affiliations:** 1 Graduate Program in Clinical and Laboratory Pathophysiology, Health Science Center / University Hospital, State University of Londrina – UEL, 60 Robert Koch Avenue – Workers’ Village, Londrina, Paraná, 86038-350 Brazil; 2 Group of Study and Research in Experimental Physiology Applied to Physical Activity (LAFEAF), Department of Physical Education, Midwestern Paraná State University, Guarapuava, PR Brazil; 3 Coordinator of the Nutrition Course, State University of Londrina – UEL, Londrina, Paraná Brazil; 4 Department of Physical Therapy, Nicole Wertheim College of Nursing & Health Sciences, Florida International University, Physical & Occupational Therapy in Geriatrics, Miami, USA

**Keywords:** Women health, Macronutrients, Nutrition

## Abstract

**Background:**

Metabolic syndrome (MetS) is a group of diseases characterized by insulin resistance. MetS has high prevalence among women, which is impacted by food intake. MetS is related to high level of inflammation; however, the impacts of whole diets on biochemical and anthropometrical markers and the effects on MetS need to be further elucidated. In this case, the objective of this study was to assess the relationship between food intake, biochemical and anthropometrical markers in women with MetS.

**Methods:**

This is a cross-sectional study, in which 1 hundred and 22 women participated in the assessment of biochemical (glycated hemoglobin, glycaemia, insulin, uric acid, total cholesterol, HDL-c, LDL-c, triglycerides, C-reactive protein) and anthropometrical (body mass, height, waist circumference - WC) variables. Participants also performed blood pressure and 24-hour dietary recall assessments. Out of the 122 participants, 44 (36%, age: 59 ± 11 years) had MetS and were included in the analysis.

**Results:**

The consumption of monounsaturated fats had direct relationship with glycaemia (b = 7.48), whereas the consumption of fibers had inverse relationship with body mass (b = − 0.71) and WC (b = − 0.56).

**Conclusion:**

The intake of monounsaturated fats and fiber was related to higher blood sugar levels and lower body mass and WC, respectively. These relationships elucidate and highlight the significance and importance of adequate diet in women with MetS.

**Supplementary Information:**

The online version contains supplementary material available at 10.1186/s12905-022-02010-7.

## Introduction

There is a relationship between visceral adiposity and diabetes mellitus, and both are related to atherosclerosis [1, 2]. In 1977, the combination of obesity, diabetes mellitus, and other clinical conditions was named metabolic syndrome (MetS) [3, 4]. Many definitions have been proposed, highlighting visceral adiposity and insulin resistance causing metabolic abnormalities [5]. Different criteria have been used to classify MetS [6–9]. In 2009, to facilitate the comparison between studies, a group of researchers proposed a common definition considering changes in fast glycaemia, hypertension, hypertriglyceridemia, low high-density lipoprotein, or the use of drugs to control any of these factors, and the presence of visceral obesity [10].

There are different interventions to reduce the risk of MetS [9]. Changes in lifestyle and eating habits are important, including controlling caloric intake and the intake of saturated fat, cholesterol, salt, and sugar. Increased consumption of fruits, vegetables, and whole grains are also recommended [11]. The risk of developing MetS is significantly affected by inadequate food consumption. In addition to food choices, food consumption patterns are also important. Identifying a diet in which individuals with MetS are able to adhere to, with moderate consumption of macronutrients, is critical [11, 12]. Carbohydrate consumption influences glycaemia, lipidic profile, inflammatory markers, and insulin resistance [13, 14]. Metabolic markers are used for clinical decisions asthey provide essential information for preventing, diagnosing and treating these diseases [15, 16]. To prescribe or guide an individualized diet, it is necessary to consider the metabolic markers for better prescription. Anthropometric measurements and food consumption patterns are also important to manage health, eating, and nutritional conditions [17].

MetS increases systemic inflammation, which differs from others inflammation processes because it involves the immunological system and impacts metabolic homeostasis. These impacts increase inflammatory markers, reduce daily energetic expenditure, promote disfunction in β cells from the pancreas, and increase hepatic and cardiovascular diseases [18, 19].

The influence of dietary habits on the control or prevention of many diseases is well known. However, more information is needed to elucidate the role of nutritional habits on inflammatory markers in people with MetS [9, 11]. Moreover, there is a gap in literature related to the impact of dietary components on different biochemical markers in individuals with MetS [20]. Furthermore, there is still a need for information regarding the impact of diets on MetS components [21]. Considering the scarcity of studies in this area, this study will provide knowledge regarding the potential effects of dietary habits on MetS and promote evidence-based practice. Therefore, this study aimed to assess the relationship between dietary components, biochemical markers, and anthropometric variables in women with MetS. The hypothesis was that adequate dietary intake would be associated with better biochemical profile and anthropometric markers.

## Methods

### Participants

This is a cross-sectional study of women attending the Multi-professional Outpatient Women’s Health Clinic at the University Hospital. One hundred and twenty-two patients were invited to participate during the years of 2018 and 2019. Patients received information about the study objectives and procedures and signed the informed consent form. The research was approved by the local ethics committee (protocol No. 2.837.313). This manuscript follows the EQUATOR network guidelines.

Participants filled a questionnaire (personal and clinical information) (Supplementary file 1) and anthropometric measurements, reported their 24-h dietary intake, and provided blood samples for biochemical analyses. The inclusion criterion was having metabolic syndrome (MetS) based on assessments, and the exclusion criteria were having other acute or chronic diseases, renal impairment, neurological diseases or cancer, pregnancy or lactation. Women with uncommon dietary patterns before 24 h of data collection were also excluded from the research.

The diagnosis of MetS was based on three or more of the following characteristics: i) waist circumference (WC) ≥ 88 cm; ii) HDL < 50 mg / dL or using antilipemic; iii) TG ≥ 150 mg/dl or using medication for dyslipidemia; iv) systolic blood pressure (BP) ≥ 130 and diastolic ≥85 mm / Hg or using antihypertensive drugs; v) Fasting venous glycaemia (FVG) ≥ 100 mg/dl or using hypoglycemic medication [10]. Of the 122 women evaluated, 44 (36%) had MetS and were included in the analyses. The high prevalence of MetS may be explained by the fact that participants were recruited from a clinical setting where they were receiving care for different health conditions, including MetS and its complications.

### Anthropometry

Body mass was measured with the subject standing barefoot and wearing light clothes using calibrated scale with 0.1 kg precision. Height was measured using stadiometer with 0.1 cm accuracy. Participants stood barefoot, with feet together, eyes gazing at the horizon without tilting or extending the head. BMI was calculated and was adopted the classification of the WHO reference standard [BMI: (weight/height^2^)] [22] for adult women and Lipschitz [23] for women aged 60 years or over. WC was measured at the midpoint between the costal arch and the iliac crest using inextensible measuring tape [22].

### Biochemical analysis

Blood samples were collected after a 12-hour fasting period in tubes without anticoagulant (to obtain serum) with separating gel; one tube containing sodium fluoride for blood glucose determination, and two tubes containing EDTA as anticoagulant and preservative. Plasma and serum were aliquoted and stored in freezer at − 80 °C (Indrel®) until tests were carried out.

Serum C-reactive protein (CRP) levels were determined by nephelometry (Dade-Behring), ELISA methodology using commercial kits with up to 5.00 mg / L of a reference value. Analyses of total cholesterol (CT), HDL cholesterol, Triglycerides (TG), uric acid, glucose, and glycated hemoglobin (HbA1c) were performed in biochemical autoanalyzer (Dimension-Siemens®), using Siemens kits. LDL-c was calculated using the following formula [24]:

$$LDL-c= Cholesterol- HDL-c-\left( Triglycerides/5\right).$$

Fasting insulin levels were determined by microparticle immunoassay enzyme on the AXSYN equipment (ABBOTT).

Insulin resistance and beta-cell function were estimated from calculations of the HOMA-IR index [25].

$$HOMA- IR= fasting\ blood\ glucose\ \left( mmol/L\right)\ x\ fasting\ insulinemia\ \left( mU/L\right)/22.5$$

The cutoff points for laboratory tests were in accordance with those already established, with the respective values for each test being considered acceptable. CRP (≤5 mg/dL), CT (< 200 mg/dL), HDL (> 50 mg/dL), LDL (< 130 mg/dL), TG (< 150 mg/dL), urid acid (≥2,6 - ≤ 6 mg/dL), glucose (≤ 70 - < 100 mg/dL), HbA1c (≤4,8 - ≤6%), insulin (≤2,7 - ≤10,4 μU/mL) and HOMA-IR (< 2,5%).

### Dietary intake assessment

Dietary intake was determined using a 24-h recall [26] with the aid of the Dietbox® software to define and quantify all the food and beverages consumed in the 24-hour period before the interview [27]. The 24-hour recall has good reproducibility with correlation coefficients between 0.36 to 0.69 and weighted Kappa over 0.4. The validity has correlation coefficients between 0.21 and 0.74 [28].

Information on dietary intake was obtained regarding the consumption of proteins, carbohydrates, sugars, fibers, total fats, polyunsaturated fats, monounsaturated, saturated, trans, cholesterol, fibers, and micronutrients. The researcher encouraged participants to remember the food intake with detailed description of foods and amounts during the interview. The researcher also revised the information at the end of the interview.

To characterize food intake as adequate or not, the total intake of macronutrients follows recommendations of the Updated Brazilian Guideline on Dyslipidemia and Atherosclerosis Prevention of the Brazilian Society of Cardiology [29].

### Statistical analysis

Sample characteristics were presented as means and standard deviations (SD). The Shapiro-Wilk test was used to verify data normality. Food consumption was presented by total amounts or daily percentages. The relationship between food consumption, anthropometrical and biochemical variables was assessed using multiple linear regression controlled by medications (hypoglycemic, hypolipemic, and insulin or similar), age and other independent variables. Regressions were expressed as regression coefficients (β) and standard errors (SE); and for variables that presented significance, confidence intervals (CI) were also presented. Residual analyses were performed to verify regression adequacy and the variance inflation factor was also verified (VIF < 2.0). Significance of 5% was adopted, and data were analyzed using the SPSS software version 25.0.”

## Results

Medications were used by 98% of participants (*n* = 43) to manage MetS. A total of 61% (*n* = 27) used oral hypoglycemic drugs, and 32% (*n* = 14) used insulins and analogues. Age and anthropometrical, biochemical, and nutritional markers are shown in Table 1. Blood pressure was within normal levels for MetS patients. As for body composition, 43% (*n* = 19) were overweight and 53% (*n* = 23) were obese.

**Table 1:** Age, Biochemical markers, anthropometric values, and dietary intake of women with metabolic syndrome

Variables	Mean ± SD	Adequate	
		%	n
Age (years)	59.0 ± 10.8		
**Anthropometric**			
Body mass (kg)	83.3 ± 12.7		
Heigh (cm)	155.9 ± 5.4		
BMI (kg/m^2^)	34 ± 5	3	2
Waist Circumference (cm)	109 ± 13	2	1
**Biochemical markers**			
HbA1c (%)	7 ± 1	36	16
Glycaemia (mg/dL)	133 ± 57	27	12
Insulin (μU/mL)	16 ± 9	25	11
Homa – RI index	5 ± 4	23	10
Uric acid (mg/dL)	5 ± 1	71	31
Total cholesterol (mg/dL)	188 ± 40	66	29
HDL-c (mg/dL)	45 ± 9	27	12
LDL-c (mg/dL)	112 ± 35	71	31
TGL-c (mg/dL)	179 ± 89	46	20
C-reactive protein (CRP) (mg/L)	6 ± 5	59	26
**Dietary intake**			
Energy (kcal/day)	1354 ± 485	16	7
Protein (g/day)	59 ± 30	34	15
Carbohydrate (g/day)	168 ± 60	34	15
Total fat (g/day)	52 ± 28	39	17
Saturated fat (g/day)	17 ± 12	18	8
Trans fat (g/day)	1 ± 1	27	12
Polyunsaturated fat(g/dia)	12 ± 5	48	21
Monounsaturated fat (g/day)	16 ± 11	41	18
Fiber (g/day)	13 ± 6	7	3

The energetic adequacy follow the DRI recommendation [30]. Adequate: Values in accordance to those described in methods for each category

Values expressed as mean and standard deviation

*BMI* body mass index, *HbA1c* glycated hemoglobin, *Homa (IR)* Homeostatic Model Assessment – Insulin resistance, *HDL-c* High-density lipoprotein, *LDL-c* Low-density lipoprotein, *TGL* Triglycerides

Regarding food consumption, 82% (*n* = 36), 52% (*n* = 23) and 59% (*n* = 26) presented high/inadequate consumption of saturated, polyunsaturated, and monounsaturated fats, respectively (Table 1); 66% (*n* = 29) of women did not consume adequate levels of proteins. The consumption of carbohydrates was adequate for only 34% (*n* = 29) of participants, and only 7% (*n* = 3) consumed adequate levels of fibers.

**Fig. 1:**
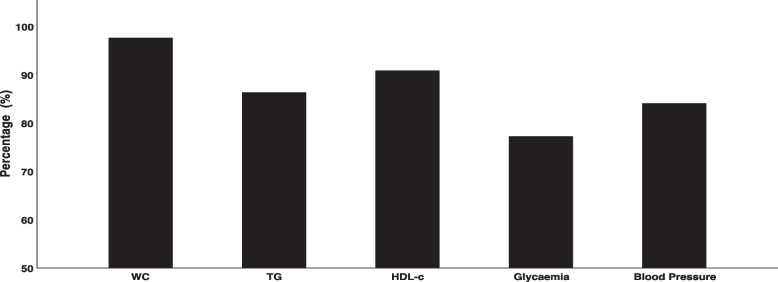
Prevalence of metabolic syndrome components of in women. Values were expressed as percentage; WC: Waist Circumference ≥ 88 cm; TG: Triglycerides ≥150 mg/dl or using medication for dyslipidemia; HDL-c: High Density Lipoproteins < 50 mg/dL or using antilipemic drugs; Glycaemia: Fasting venous glycaemia (FVG) ≥ 100 mg/dl or when using hypoglycemic drugs; Blood Pressure: systolic blood pressure (BP) ≥ 130 and diastolic ≥85 mm / Hg or when using antihypertensive drugs

The prevalence (%) of disorders related to MetS among participants is shown in Fig. 1. WC was high (> 88 cm) in 98% (*n* = 43), 91% (*n* = 40) had low HDL-C or used drug treatment to reduce HDL-c, 84% (*n* = 37) had hypertension or used antihypertensives, and 86% (*n* = 39) had high TG concentration or used medication for dyslipidemia.

Table 2 shows the relationship between food consumption and biochemical variables. The relationship between consumption of monounsaturated fats and glycemic values was significant (β: 7.48; CI:1.24–13.72; *P < 0.05*). The consumption of other macronutrients, on the other hand, did not show significant relationship with biochemical variables.

**Table 2:** Relationship between dietary intake and biochemical markers of women with metabolic syndrome

	Kcal total	Protein	Lipids	CHO	Fat		Fiber
					Poli	Mono	
HbA1c	0.001 (0.01)	0.001 (0.03)	−0.05 (0.06)	− 0.001 (0.02)	0.02 (0.04)	0.11 (0.07)	0.01 (0.03)
Glycaemia	0.13 (0.29)	−0.24 (1.29)	−3.85 (2.85)	−0.48 (1.11)	0.62 (2.2)	**7.48 (3.06)**	−0.46 (1.33)
Insulin	0.002 (0.05)	0.041 (0.23)	−0.063 (0.52)	0.02 (0.20)	0.5 (0.39)	0.14 (0.6)	0.07 (0.24)
Homa RI	−0.001 (0.02)	0.02 (0.08)	−0.10 (0.18)	0.01 (0.07)	0.16 (0.14)	0.3 (0.21)	−0.02 (0.08)
Uric acid	0.003 (0.01)	0.01 (0.04)	0.001 (0.08)	−0.01 (0.03)	−0.07 (0.06)	− 0.09 0.1)	0.05 (0.04)
CT	0.19 (0.23)	1.01 (1.02)	1.59 (2.27)	0.76 (0.88)	−1.39 (1.72)	−1.08 (2.63)	− 1.4 (1.06)
HDL	−0.03 (0.06)	0.08 (0.25)	0.31 (0.55)	0.14 (0.22)	0.09 (0.42)	−0.21 (0.64)	−0.24 (0.6)
TGL	−0.14 (0.54)	1.39 (2.41)	1.6 (5.33)	0.14 (2.08)	−1.31 (4.05)	−1.7 (6.17)	2.24 (2.5)
CRP	0.003 (0.03)	0.02 (0.12)	−0.16 (2.63)	0.002 (0.10)	0.11 (0.2)	0.29 (0.31)	−0.004 (0.1)

Values were expressed in Beta coefficient and standard error (SE)

Bold letter: *P* < 0.05

*HbA1c* glycated hemoglobin, *Homa (IR) Homeostatic Model Assessment –* Insulin resistance, *CT* Total cholesterol, *HDL-c* High-density lipoprotein, *LDL-c* Low-density lipoprotein, *TGL* Triglycerides, *CRP* C-reactive protein, *CHO* Carbohydrates

Table 3 shows the relationship between food consumption and anthropometric variables. Inverse relationship was observed between fiber consumption and body mass (β: -0.71; CI: − 1.34 - -0.07; *P < 0.05*) and waist circumference (β: -0.56; CI: − 1.21 – -0.01; *P < 0.05*).

**Table 3:** Relationship between dietary intake and anthropometric measurements of women with metabolic syndrome

	Kcal total	Protein	Lipids	CHO	Fat		Fiber
					Poly	Mono	
Body mass (kg)	−0.04 (0.06)	0.26 (0.3)	−0.01 (0.61)	0.24 (0.26)	0.08 (0.50)	0.82 (0.78)	**−0.71 (0.32)**
BMI (kg/m^2^)	−0.02 (0.03)	0.12 (0.12)	0.11 (0.10)	−0.05 (0.24)	0.49 (0.31)	0.13 (0.2)	−0.14 (0.12)
WC (cm)	−0.04 (0.07)	0.3 (0.30)	0.3 (0.26)	−0.04 (0.62)	0.78 (0.79)	0.30 (0.51)	**−0.56 (0.32)**

Values were expressed in Beta coefficient and standard error (SE)

Bold letter: *P* < 0.05

*WC* Waist circumference, *CHO* Carbohydrates

## Discussion

This research aimed to assess the relationship between food consumption, biochemical markers, and anthropometric values in women with MetS. Significant relationship was observed between consumption of monounsaturated fat and fast glycaemia, and inverse relationship between fiber consumption, body mass and waist circumference.

Recent studies have emphasized the impact of macronutrient intake on the energetic biomarkers related to metabolic health; genetic effects are more powerful when healthy diets are consumed [31]. Gene/macronutrient interactions modulate the risk of obesity and metabolic diseases [32]. In the present study, high ingestion of fats and low ingesting of fibers was observed. Direct relationship between monounsaturated fat intake and high fast glycaemia was also observed. The consumption of monounsaturated fats can be related to the high consumption of red meat and dairy products; and distinct cardiometabolic effects are related to the consumption of mono and polyunsaturated fats [33]. Jiao et al. [34] found positive association between monounsaturated fat and mortality.

Consumption of red meat, especially processed meat, was related to increase in diabetes. In contrast, the intake of milk, cheese, and yogurt showed neutrality over cardiometabolic effects. Vegetables that provide monounsaturated fat had positive cardiometabolic effects [33, 35, 36]. The 24-h dietary recall confirmed the choice of unhealthy sources of monounsaturated fat such as processed meats and cheese, which have high amounts of saturated fat with postprandial effects, promote the store of fat (lipogenesis), increase inflammatory processes and glycaemia [37, 38].

Another important factor for metabolic homeostasis is the intestinal microbiota, which is dependent on alimentary residues such as fiber, for its surveillance and metabolism [39]. Moreover, the intestinal microbiota provides high butryogenesis rates, with epigenetic and immunomodulatory effects on other organs of the body [40]. This information could help understanding the relationship between high fiber intake and reduction in many pathologies, including metabolic diseases [41–45]. It is important to highlight the low fiber intake found in the present study (13 g/day), which was 50% less than the recommended values of 26 g/day [29].

The analysis of dietary intake and anthropometrics measurements revealed inverse relationship between fiber intake and waist circumference. This result corroborates other studies. Liu et al. [46] evaluated 74.091 nurses over 12 years and observed that those with high fiber intake gain less weight than their peers. Furthermore, overweight and obese women have lower intake of fruits and vegetables [47]. Dietary fiber intake is inversely related to body mass, BMI and WC [48–50]. Fiber consumption is critical to promote weight loss in obese or overweight subjects [49, 50]. The most prevalent MetS factor in the present study was high WC (98% > 88 cm). Fibers prevent obesity by supporting the intestinal microbiota, decreasing the absorption of nutrients [51], inhibiting appetite [52, 53], and regulating homeostasis [54, 55].

This study demonstrates that small changes in eating habits, as decrease in monounsaturated fat intake and increase in fiber intake, could reduce the risk factors for women with MetS. While monounsaturated fats have relationship with glycaemia, fibers have inverse relationship with body mass and WC. The data found in this study enhance the importance of dietary fiber intake since it could promote beneficial effects on the anthropometric profile.

The present study has some limitations such as the small sample size, only one dietary intake assessment, and lack of data on physical activity levels. The 24-h dietary recall is subject to errors in the representation of the actual dietary intake. However, there is no gold standard method available for researchers to evaluate dietary intake [56, 57]. Even applied only once, this recall search estimates the absolute intake instead of relative intake through its open structure [58].

## Conclusion

The intake of monounsaturated fats and fiber were associated to higher blood sugar levels and low body mass and waist circumference, respectively. These relationships highlight the importance of dietary intake in MetS management.

The present study increases the understanding of the relationship between dietary habits, biochemical and anthropometric markers in women with MetS. The findings indicate that the consumption of fibers is important to the control of visceral obesity in women with MetS. Further longitudinal studies are necessary to evaluate the cause-effect relationship between dietary intake and its effects on physiological and anthropometrical markers in people with MetS.

## Supplementary Information

**Additional file 1.**

## Data Availability

The datasets used and analyzed during the current study are available from the corresponding author on reasonable request.

## Abbreviations

BMI: Body Mass index
BP: systolic blood pressure
CRP: C-reactive protein
CT: Total cholesterol
CI: 95% confidence interval
FVG: Fasting venous glycaemia
HbA1c: Glycated hemoglobin
HDL-c: High-density lipoprotein
LDL-c: Low-density lipoprotein
MetS: Metabolic syndrome
SD: Standard deviations
SE: Standard errors
TG: Triglycerides
WC: Waist circumference
β: Regression coefficients
