# Supplementary material for: Impacts of food consumption on biochemical markers and anthropometric variables of women with metabolic syndrome
Source: BMC Womens Health. 2022 Oct 26;22:423. doi: 10.1186/s12905-022-02010-7 (PMC9598031; doi:10.1186/s12905-022-02010-7)
Supplement: Supplementary file 1 — Additional file 1. [file 12905_2022_2010_MOESM1_ESM.pdf]

CASE HISTORY

IDENTIFICATION NUMBER____
Date: ___/___/___

Identification:

Name: ____
Patient's record number: ____________
Birth day: ___/___/___ *Age:*________
*Education:*____

Clinical data:

Clinical diagnosis: ____
Metabolic Syndrome diagnosis: ____
Clinical History: ____
Signals and symptoms: ____
____
*Musculo skeletal Pain: (   ) Não (   ) Sim*___________________________________
Drugs in Use: ____
Antioxidants: ____
Tabacoo use: (   ) no (   )yes, Frequency: ___________________________________
Alcoholism: (   ) no (   )yes, Frequency:______________________________________
Blood Pressure: ____

Anthropometric Data

Weight _____kg; Height: ______m;
BMI: ______kg/m^2; Classification: _________________;
Waist Circumference: ____cm

Nutritional Data

Recently weight alterations: ( ) no ( )yes,
Observations: ____
Additional observations in relation to the eating habits:____
____
Hydric ingestion/day:_________________ Recommendation:___________________
Food Allergic: (   ) No   (   ) yes, Observation:________________________________
